# Childhood trauma is associated with early-onset but not late-onset suicidal behavior in late-life depression

**DOI:** 10.1017/S1041610223000662

**Published:** 2023-08-29

**Authors:** Ya-Wen Chang, Morgan Buerke, Hanga Galfalvy, Katalin Szanto

**Affiliations:** 1 Department of Psychiatry, University of Pittsburgh School of Medicine, Pittsburgh, PA, USA; 2 Department of Psychology, University of Southern Mississippi, Hattiesburg, MS, USA; 3 Department of Psychiatry, Columbia University College of Physicians and Surgeons, New York, NY, USA; 4 Department of Molecular Imaging and Neuropathology, New York State Psychiatric Institute, New York, NY, USA

**Keywords:** trauma, suicide, depression, childhood abuse, late-life

## Abstract

**Objectives:**

To examine the relationship between childhood traumatic experiences and early and late-onset suicidal behavior among depressed older adults.

**Design:**

Cross-sectional study.

**Setting:**

Inpatient and outpatient psychiatric services in Pennsylvania.

**Participants:**

Our sample included 224 adults aged 50 + (M ± SD = 62.5 ± 7.4) recruited into three depressed groups: (1) 84 suicide attempters, (2) 44 suicide ideators, and (3) 58 non-suicidal comparisons, and a non-psychiatric healthy comparison group (*N* = 38).

**Measurements:**

The Childhood Trauma Questionnaire measured experiences of childhood trauma such as emotional abuse, physical abuse, emotional neglect, physical neglect, and sexual abuse.

**Results:**

Attempters were separated into early- and late-onset based on age of first attempt using a statistical algorithm that identified a cutoff age of 30 years old. Overall, we found group differences in emotional and physical abuse and neglect in both genders and sexual abuse in females, but not in males. Early-onset attempters experienced more childhood emotional abuse and neglect than late-onset attempters and were more likely to have experienced multiple forms of abuse. They also experienced more emotional abuse and neglect than all comparison groups. Consistently, early-onset attempters more often met criteria for current or lifetime PTSD relative to late-onset attempters and most comparison groups. Late-onset attempters had similar levels of childhood trauma as other depressed groups.

**Conclusions:**

Our study reaffirms that there are distinct pathways to suicidal behavior in older adults based on their age of first suicide attempt and that trauma experienced in childhood has long-lasting emotional and behavioral consequences, even into late life.

## Introduction

Negative early life events are well-known for having an impact on functioning years after the events occur. For instance, it has been well established that traumatic early life experiences can adversely impact long-term psychopathology, leading to depression and suicidal behavior into adolescence and young adulthood (Barbosa *et al*., 2014; Dias de Mattos Souza *et al*., 2016; Dube *et al*., 2001; Erol *et al*., 2013; Harford *et al*., 2014; Ng *et al*., 2018). However, it is unclear how long early life trauma can impact psychopathology, and whether this same relationship extends even into late life. Even those who study the relationship between childhood trauma and later suicide often conduct their studies in samples of mixed ages, including very few participants over the age of 60, and neglecting to include the oldest age groups (i.e. those 75 years and older; Angelakis *et al*., 2019; Barbosa *et al*., 2014; Behr Gomes Jardim *et al*., 2018; Bruffaerts *et al*., 2010; Dias de Mattos Souza *et al*., 2016; Draper, 2015; Ng *et al*., 2018; Radford *et al*., 2017). As older adults, especially those 85 and older, remain at the highest risk for death by suicide of any age group (Curtin *et al*., 2022), it is important to examine how negative life experiences from early life can combine with events unique to later life to predict late-life suicidal behavior.

The experience of early life trauma has been separated into two categories: childhood abuse, or maltreatment or assault resulting in injury or harm to the well-being of a child, and childhood neglect, or a caregiver’s inability to provide for a child’s fundamental physical or emotional needs (Bernstein *et al*., 1997, 2003). Importantly, abuse and neglect have distinct influences on cognitive, emotional, and neurodevelopmental processes (Turecki *et al*., 2012). McLaughlin and Sheridan (2016) proposed a dimensional model stating that exposure to different types of early adversities is associated with numerous neurodevelopmental outcomes, where abuse is associated with salience processing, aversive learning, and emotion regulation, and experiences of neglect are linked to cognitive development and executive functioning. As executive functioning can decline with age, it may lead those with childhood trauma to have compounded risk for suicidal behavior in old age (Szanto *et al*., 2018, 2020).

In addition, experiences of childhood abuse and neglect can be further broken down into emotional and physical subtypes, where abuse can also include childhood sexual abuse (Bernstein *et al*., 2003). Evidence suggests that different types of childhood abuse and neglect may differentially impact risk for suicidal behavior. For example, physical and sexual abuse have been the most consistent subtypes of childhood trauma experiences linked to suicidal behavior across the lifespan (Angelakis *et al*., 2019; Behr Gomes Jardim *et al*., 2018; Bruffaerts *et al*., 2010; Draper, 2015; Ng *et al*., 2018; Radford *et al*., 2017). Suicide risk is also elevated in young adults who have experienced emotional abuse and neglect in childhood (Barbosa *et al*., 2014; Dias de Mattos Souza *et al*., 2016; Erol *et al*., 2013), but the few studies including older adults, have not found this same relationship (Behr Gomes Jardim *et al*., 2018). In contrast, most studies have not found any relationship between physical neglect and suicide. Therefore, physical and sexual abuse may be expected to have the strongest association with suicide in late life; however, further research is necessary to confirm this relationship.

When studying suicide in older adults, it is crucial to examine risk factors for suicide that consider the real-life complexities that exist within this heterogenous group (Szanto *et al*., 2018) to determine individualized risk factors and develop tailored interventions. One well-established difference in risk profiles is based on when a suicidal older adult made their first suicide attempt. For instance, older suicide attempters who have their first incidence of suicidal behavior early in life whilst still being suicidal into late life (early-onset attempters) often have more attempts, but with lower lethality than those who do not make their first attempt until late life (late-onset attempters; Szücs *et al*., 2020). Experiencing trauma in childhood can also result in the development of other early-onset psychopathology, such as early-onset depression and borderline personality disorder, which are further associated with an increased risk of future suicidal behaviors (Bozzatello *et al*., 2021; Wielaard *et al*., 2018).

Consistent with this idea, childhood trauma has been found to predict earlier onset of suicidal behaviors in younger samples (Bruffaerts *et al*., 2010; Roy, 2004; Slama *et al*., 2009), but it is unclear whether childhood trauma can interact with late-life experiences to lead to long-lasting emotional and behavioral consequences decades after the trauma occurred. Additionally, as we know that other risk factors for suicide, such as impulsivity and borderline traits, decrease with age (Buerke *et al*., 2021; Steinberg *et al*., 2008; Stevenson *et al*., 2003), the question remains whether the effect of experiencing childhood trauma would similarly decline over the decades.

The present study aimed to investigate the relationship between various types of childhood trauma and early and late-onset suicidal behavior among depressed older adults. Our previous findings have indicated that early-onset suicide attempters share characteristics known to be associated with suicide attempts in younger age, such as personality traits, exposure to suicide in family and/or friends, and suboptimal decision-making patterns (Kenneally *et al*., 2019; Perry *et al*., 2021; Szücs *et al*., 2020), while memory and broader cognitive impairments are associated with late-onset attempts (Gujral *et al*., 2020).

Additionally, previous studies have shown that individuals with depression across various age groups have a higher prevalence of childhood abuse (Lindert *et al*., 2014). To explore this relationship further, we included a number of comparison groups; two with psychopathology (those with depression and ideation, those with depression but no ideation) and one without (non-psychiatric comparisons), to determine whether childhood trauma’s effect extends above and beyond the effect of suicidal ideation and depression. We hypothesized that those whose first attempt occurred early in life would be more likely to experience each form of childhood trauma than those whose first attempt occurred in late life. We also hypothesized that all depressed groups would be more likely to suffer from each form of childhood trauma than the healthy comparison group and that those with ideation would have experienced more trauma than those who were depressed with no ideation. To explore confounders that may better explain the relationship between childhood trauma experiences and suicidal behaviors, we also compared clinical characteristics, including borderline traits, impulsivity, cognition, and lifetime/current psychiatric disorders in our sample.

## Methods

### Sample

Two hundred and twenty-four participants aged 50 and older (range: 50–81; *M* = 62.5, SD = 7.4) were recruited between January 2005 and February 2021 into a case-control longitudinal study, the Longitudinal Research Program for Late Life Suicide (Szanto *et al*., 2015). All study procedures were approved by the University of Pittsburgh Institutional Review Board (IRB0407166), and all participants provided written informed consent.

Psychiatric patients were recruited from various sources including inpatient psychiatric units, outpatient clinics, and the community, whereas the non-psychiatric comparison group was exclusively recruited from the community through the University of Pittsburgh Pitt + Me registry and community advertisement. Participants were assessed for current and lifetime psychiatric illness, including substance abuse, depressive disorders, and anxiety disorders, using the Structured Clinical Interview for DSM-IV (SCID; First *et al*., 1995). Exclusion criteria included (1) history of neurological disorders, delirium, psychosis, mania, or dementia; (2) severe cognitive impairment as assessed by a score of 22 or lower on the Mini-Mental State Examination (MMSE; Folstein *et al*., 1975); (3) active withdrawal from substances; or (4) ECT treatment within the past 6 months.

The sample was recruited into four groups: depressed suicide attempters (*N* = 84), depressed suicide ideators (*N* = 44), a non-suicidal depressed comparison group (*N* = 58), and a non-psychiatric healthy comparison group (*N* = 38). *Suicide attempters* made either (1) at least one recent suicide attempt (i.e., self-injurious act with intent to die) within a month of entry into the study or (2) a suicide attempt at any time prior to enrollment with current active suicidal ideation as assessed by the Beck Scale for Suicidal Ideation (SSI; Beck *et al*., 1979). *Suicide ideators* had no lifetime history of suicidal behavior and were required to endorse current active suicidal ideation assessed using the SSI (Beck *et al*., 1979). The *non-suicidal depressed comparison group* had no lifetime history of self-injurious or suicidal behavior and no lifetime history of suicidal ideation. All depressed groups were diagnosed with current unipolar depression without psychosis as determined by the SCID and scored a 14 or higher on the 17-item Hamilton Rating Scale for Depression (HRSD; Hamilton, 1960). The *non-psychiatric healthy comparison group* had no lifetime history of psychiatric disorders (including substance abuse) based on the SCID. The healthy comparison group also had no lifetime history of suicidal ideation, any self-injurious act, or suicidal behavior.

### Instruments

#### Childhood trauma questionnaire

The Childhood Trauma Questionnaire – Short Form (Bernstein *et al*., 2003) is a 28-item self-report measurement that evaluates the following forms of abuse and neglect experienced in childhood or adolescence: emotional, physical, and sexual abuse, as well as emotional and physical neglect. Responses are endorsed on a scale from 1 = “never true” to 5 = “very often true,” with higher scores indicating more severe traumatic experiences on each of the subscales. Emotional abuse was defined as humiliating or degrading behaviors and attacks on well-being of a child (e.g., “I thought that my parents wished I had never been born”). Physical abuse is described as any physical assault that caused injuries (e.g., “I got hit so hard by someone in my family that I had to see a doctor or go to the hospital”). Sexual abuse is defined as sexual contact/conduct between a minor (younger than 17 years of age) and an older person (at least 5 years older than the child; e.g., “Someone tried to touch me in a sexual way or tried to make me touch them”). Emotional neglect is a relationship pattern in which caretakers persistently disregard, ignore, or invalidate the affectional needs of a child (e.g., reversed; “There was someone in my family who helped me feel important or special”). Physical neglect was defined as a caregiver’s inability to meet a child’s fundamental physical requirements, such as food, clothing, housing, safety, and health care, or poor parental supervision (e.g., “My parents were too drunk or high to take care of the family”).

#### Other descriptive and clinical measures

*Suicide history* was assessed by evaluating the number of lifetime suicide attempts and the age of first attempt.

*Beck Lethality Scale* (BLS; Beck et al., 1975) was used to measure lethality of the most severe attempt based on the severity of its medical consequences, which ranged from 0 (none or little) to 8 (attempt resulting in death).

*Scale of Suicidal Intent* (SIS; Beck et al., 1974) was used to assess the degree of planning of the attempt and the participant’s intent to execute the suicidal behavior. For the present study, we report the planning subscale and total score of the SIS.

*Scale of Suicidal Ideation* (SSI; Beck et al., 1979) was used to evaluate the severity of current and worst lifetime suicidal ideation. This assesses the frequency and length of suicidal thoughts, as well as the factors that potentially discourage people from attempting suicide.

*Hamilton Rating Scale for Depression* – *17 Item* (HRSD; Hamilton, 1960) was used to assess depression severity, where higher scores indicate more severe depression. We removed the suicidal ideation item from analysis to avoid inflating the overall scores of suicidal individuals.

*Structured Clinical Interview for DSM-IV* (SCID; First *et al*., 1995) was a semi-structured interview used to assess for DSM-IV diagnoses and age of first episode. Current psychiatric diagnosis was assessed over the past month.

*Personality Assessment Inventory* – *Borderline subscale* (PAI-BOR; Morey, 2014) measures borderline personality traits.

*Barratt Impulsiveness Scale* (BIS-11; Patton et al., 1995) measures attentional, motor, and nonplanning impulsivity.

To assess cognitive dysfunction, we used three neuropsychological assessment tools: the *MMSE* (Folstein et al., 1975) and the *Mattis Dementia Rating Scale* (Mattis, 1988) assessed for global cognitive ability and the *Executive Interview* (Royall et al., 1992) assessed for executive function.

### Analytic strategy

**Figure 1.**
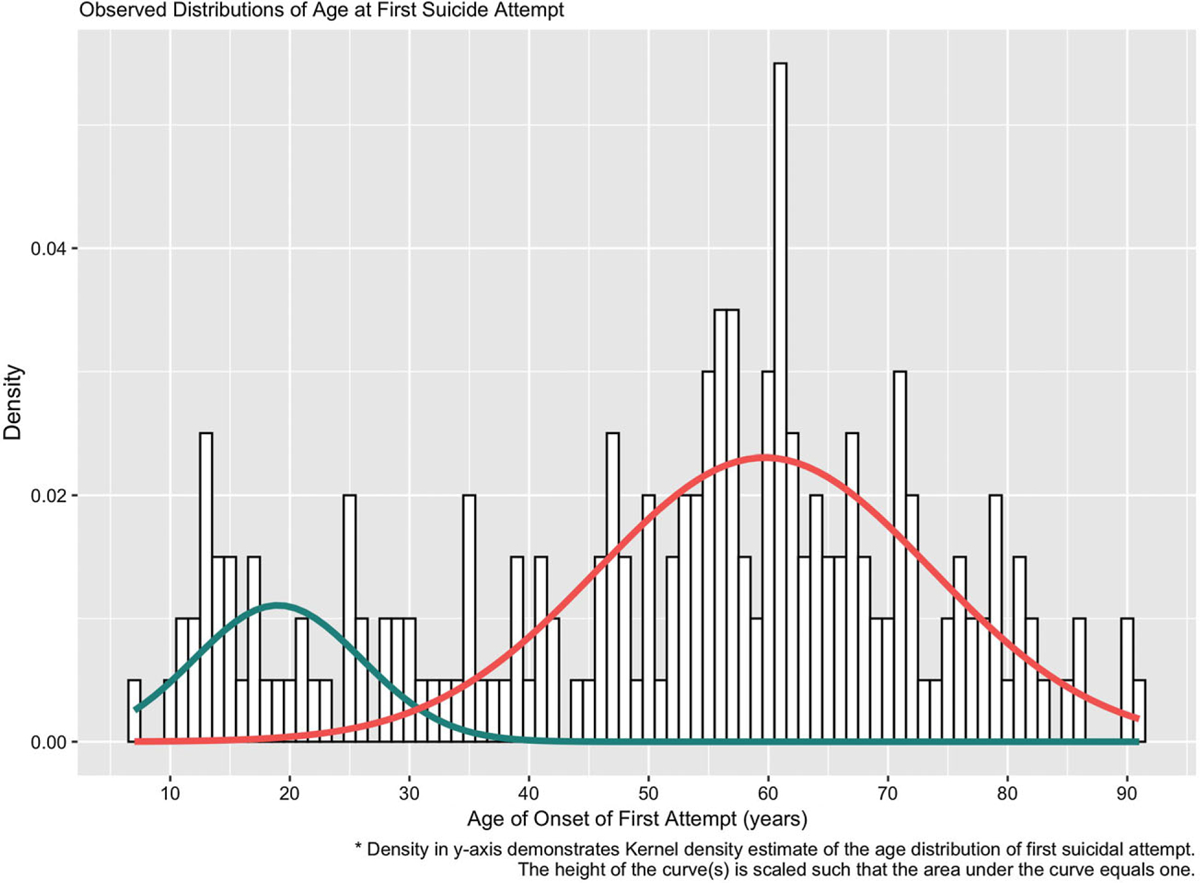
Observed distributions of age of onset of first suicide attempt: density in y-axis demonstrates kernel density estimate of the age distribution of first suicidal.

All analyses were performed using R version 4.0.2 (R Core Team, 2021). The distribution of age of the first suicide attempt was graphed using a histogram and was found to display a bimodal distribution (see Figure 1). To identify component populations, we applied Gaussian mixture modeling (GMM) using the “mixtool” library (Benaglia *et al*., 2010). This is a probabilistic model used to fit normally distributed subpopulations, which we applied to a larger sample recruited by the Pittsburgh Longitudinal Study of Late-Life Suicide (PI: K. Szanto, *N* = 589 with 200 attempters). To specifically address our research question related to childhood trauma, we then concentrated on a subset of participants who completed the Childhood Trauma Questionnaire (*N* = 224 with 84 attempters). The optimal number of components, out of a possible range of 1 to 6, was established using the bootstrap test and was found to be 2 components. Subjects were split into groups based on the age of onset cutoff value where the two density curves met (see Figure 1). Using this grouping based on the GMM-generated age-at-first-attempt cutoff, we present summary statistics alongside the corresponding characteristics for the comparison groups, namely suicidal ideators, non-suicidal depressed controls, and a healthy comparison group (see Table 1).

To test our main hypothesis regarding the relationship between childhood trauma and age of onset of suicidal behavior, we used univariate analyses to compare these attempter groups to each other and each comparison group on childhood trauma. For sexual abuse, males and females were analyzed separately, given that sexual abuse among men is more strongly associated with suicidal behavior (Baiden *et al*., 2020; Martin *et al*., 2004; Rhodes *et al*., 2011). We used one-way analysis of variance (ANOVAs) for continuous variables and chi-square tests for categorical variables. For continuous variables, outliers defined by being more than 1.5 interquartile range above the third quartile, or below the first quartile on each variable, were winsorized before testing, except for sexual abuse due to its rarity in reporting. To examine group differences for ordinal variables, variables with extremely skewed distributions or variables with different variances among groups, we performed non-parametric tests such as the Kruskal-Wallis Test in place of ANOVAs. When the group effect was significant, we conducted *post hoc* pairwise comparisons to determine pairwise differences between the five groups, using Tukey’s HSD for continuous variables, and Bonferroni for categorical or ordinal variables to adjust for multiple comparisons.

The effect size for comparing childhood trauma experiences between early-onset and late-onset suicide attempters was calculated and reported with 95% CI unadjusted for multiple comparisons. Cohen’s d effect sizes were computed for continuous outcomes using the rstatix package (Kassambara, 2023) and odds ratio (OR) for binary outcomes, with the corresponding 95% CI calculated using the Epitools package (Aragon *et al*., 2020).

Finally, to explore potential confounders of any relationships we found between childhood trauma and onset of suicidal behavior, we compared groups on borderline traits, impulsivity, cognition, lifetime/current psychiatric disorders, depression severity, and suicide characteristics using the same technique as in our main analysis.

## Results

### Early and late-onset differences

Using the cutoff score of 30 from the results of Gaussian mixture modeling, we identified two groups of suicide attempters: *Early-onset attempters* and *Late-onset attempters*, with the best division operationalized by whether their first attempt took place before/at age 30 or later. Among the 84 attempters, the early-onset group consisted of 20% of the attempters (first attempt: 7–29 years, mean = 17.5, SD = 6.4), with the late-onset group consisting of the other 80% (first attempt: 31–81 years, mean = 57.2, SD = 12.4).

### Sociodemographic characteristics

Statistics presented in Table 1 describe the demographic characteristics of the five groups. There were no differences in age, sex, race, or ethnicity. All depressed groups had lower income relative to the non-psychiatric healthy comparison group. The healthy and depressed non-suicidal comparison groups had higher levels of education than late-onset attempters.

### Differences in childhood trauma between groups

Overall, we found significant group differences (Table 2) in emotional abuse (*F* (4, 219) = 11.718, *p* < 0.001), physical abuse (*F* (4, 219) = 8.636, *p* < 0.001), emotional neglect (*F* (4, 219) = 9.886, *p* < 0.001), and physical neglect (*F* (4, 219) = 6.911, *p* < 0.001) in both genders and sexual abuse in females (*F* (4, 123) = 2.522, *p* = 0.045), but not in males (p > 0.05).

**Figure 2.**
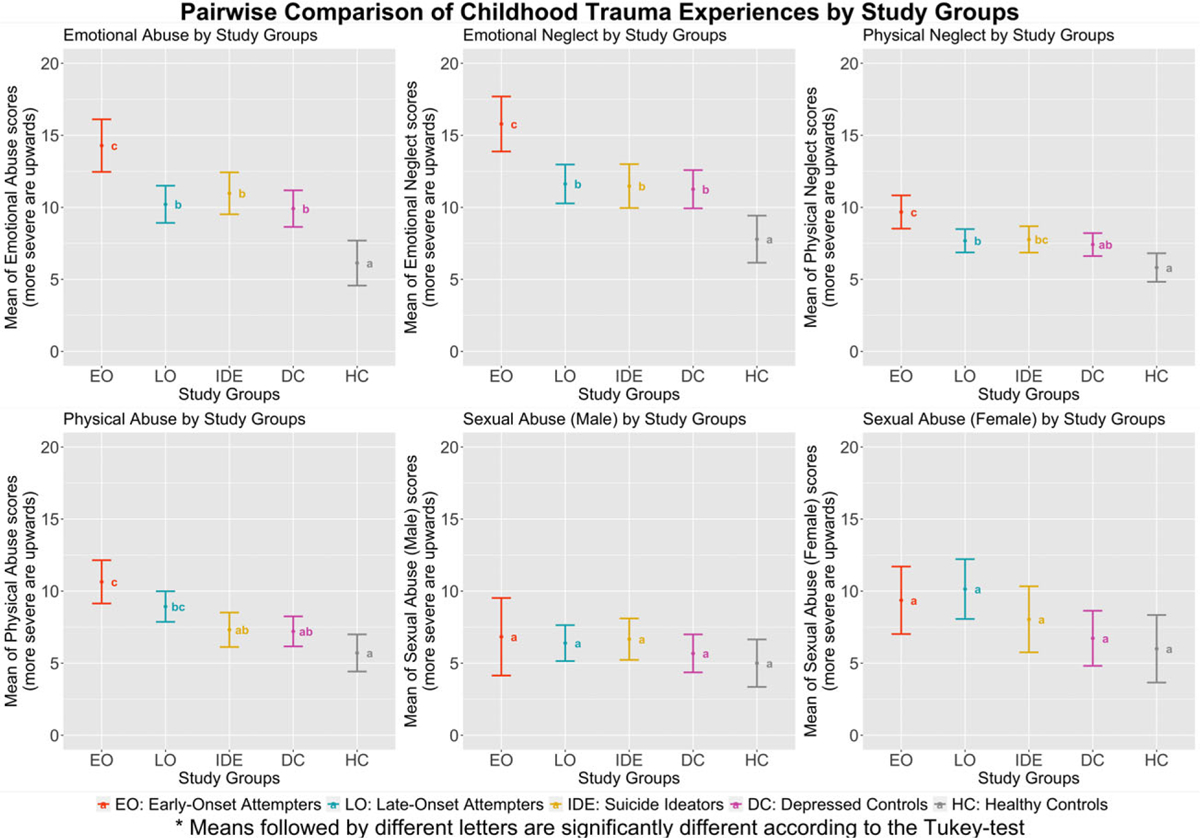
Pairwise comparison of childhood trauma experiences by group: emotional abuse, emotional neglect, physical neglect, physical abuse, and sexual abuse in males and females were assessed, with higher scores indicating higher severity. EO = early-onset attempters; LO = late-onset attempters; IDE = suicide ideators; DC = depressed controls; HC = healthy controls. Early-onset attempters experienced more physical abuse than suicide ideators, depressed controls, and healthy controls; late-onset attempters only experienced more physical abuse than healthy controls. Both attempter groups reported significantly more sexual abuse than healthy controls. The points indicate mean scores, the whiskers indicate standard deviations. Groups labeled with non-identical letters are significantly different after Tukey’s adjustment.

The pairwise comparisons (Figure 2; Table 2) indicated that early-onset attempters experienced more emotional abuse (*d* = 0.75, 95% CI: [0.30–1.26]), emotional neglect (*d* = 0.76, 95% CI: [0.28–1.36]), physical neglect (*d* = 0.59, 95% CI: [0.10–1.12]), and overall childhood trauma (*d* = 0.77, 95% CI: [0.30–1.31]) than late-onset attempters. In addition, early-onset attempters reported a higher likelihood of endorsing at least one trauma (OR = 4.64, 95% CI: [1.53–17.89]), and multiple forms of trauma (OR = 3.44, 95% CI: [1.36–9.43]), than late-onset attempters. However, the effect sizes for physical abuse (*d* = 0.39), sexual abuse for men (*d* = 0.12), and sexual abuse for women (*d* = − 0.11) were not significant.

Early-onset attempters also experienced more overall childhood trauma, likelihood of endorsing at least one trauma, and greater likelihood of multiple forms of trauma than all other comparison groups. In contrast, late-onset attempters were not different from other depressed groups. Further, while early and late-onset attempters did not differ on physical abuse, early-onset attempters experienced more physical abuse than comparison groups, while late-onset attempters only experienced more physical abuse than the healthy comparison group. While sexual abuse in females showed overall group differences, no differences in sexual abuse history were found between any pairs of groups in female participants.

### Potential pathways

In order to test potential pathways that explain why each group may have differed on childhood trauma, clinical characteristics were compared among the five groups (Table 3). All clinical groups reported higher borderline traits and impulsivity than the healthy comparison group, and ideators had higher borderline traits than the depressed comparison group. On cognitive variables, both attempter groups showed more impairment in overall cognition than the healthy comparison group. Late-onset attempters also had worse executive functioning (denoted by higher scores) than the healthy comparison group and lower cognitive performance than depressed controls.

No differences were found in lifetime and current substance abuse disorder between groups. Early-onset attempters were more likely to meet criteria for a lifetime anxiety disorder than depressed comparisons. Groups further differed on the presence of current anxiety disorders, with no pairwise differences between groups. Depression severity was higher in both early- and late-onset attempters than in the non-suicidal depressed comparison group. Early-onset attempters’ first depressive episode was earlier than all other depressed groups. Prevalence of PTSD was also significantly different among all clinical groups, where early-onset attempters were more likely to endorse symptoms of lifetime and current PTSD than almost all other groups, except for current PTSD in suicide ideators, and also endorsed the earliest onset of PTSD.

Finally, the three groups with suicidal ideation/behavior differed on suicide-related variables, as late-onset attempters reported higher current ideation than all other groups, and both attempter groups had higher worst-ever ideation scores than ideators. Early-onset attempters reported more lifetime suicide attempts compared to late-onset attempters. However, attempter groups did not differ in levels of intent, degree of planning, or lethality of most severe suicide attempt.

## Discussion

We examined differences in childhood trauma experiences between early and late-onset suicide attempts, with comparison groups of depressed ideators (suicidal ideation but no history of suicidal behavior), non-suicidal depressed, and non-psychiatric comparisons. As hypothesized, early-onset attempters had higher levels of overall childhood trauma, and a higher likelihood of having at least one trauma and multiple traumas, than all other groups. Early-onset attempters also experienced more emotional abuse and neglect and physical neglect than late-onset attempters, whereas late-onset attempters had similar levels of these forms of childhood trauma as non-attempter comparison groups. Specifically, there were large effect sizes for emotional abuse and neglect, and overall childhood trauma, indicating substantial differences between early- and late-onset attempters. No significant differences were found in physical and sexual abuse.

Our earlier studies used a less sophisticated, but still data-driven approach employing median splits ranging from 50 to 59 years old for early vs. late-onset suicide attempts (Gujral *et al*., 2020; Kenneally *et al*., 2019; Perry *et al*., 2021; Szücs *et al*., 2020), however, this approach is based on the a priori assumptions that the number of components is two and that the population of attempters is split equally. Parametric bootstrap testing in conjunction with GMM allowed us to identify the optimal number of components to identify two distinct peaks (see Fig. 1). This GMM approach also provided a reasonable fit to the present data based on human visual examination and provides a useful tool for explaining heterogeneity in the age of onset data, although we acknowledge that this is only an approximation to the unknown, true underlying process. The age cutoff for late-onset depression has varied in the literature but studies agree that individuals who experience depression at earlier ages tend to have a genetic predisposition for depression, while older adults who have late-onset depression have more cognitive deficits, cerebral structural abnormalities, and vascular disease that may cause their depressive symptoms (Bukh *et al*., 2011; Sekhon *et al*., 2023). The age cutoff of 30 for early-onset suicidal behavior in our study makes sense from a brain development standard, as other studies have shown that personality and brain changes stabilize around this time (Sekhon *et al*., 2023).

Our study builds upon our prior research that investigated phenotypic heterogeneity among suicide attempters based on age of first suicide attempt and described two distinct pathways for suicidal behavior in late life (Gujral *et al*., 2020; Kenneally *et al*., 2019; Perry *et al*., 2021; Szücs *et al*., 2020). Risk for early-onset attempts has been associated with similar risk factors to attempts that are reported in younger adults, with emphasis on factors such as impulsivity, decision-making deficits, personality dysfunction, and exposure to suicide in family and/or friends (Kenneally *et al*., 2019; Perry *et al*., 2021; Szücs *et al*., 2020). Our finding that early-onset suicidal behavior was associated with higher levels of childhood trauma than other depressed groups is consistent with this growing body of research as well as other findings linking childhood trauma to suicidal behavior in younger adults (Behr Gomes Jardim *et al*., 2018). As early-onset attempters were also more likely to have multiple forms of trauma, it is clear that the cumulative experience of childhood trauma is associated with risk for suicide (Allbaugh *et al*., 2017; Torchalla *et al*., 2012) that persists even into late-life.

Instead, consistent with past research on early-onset suicide attempters, experience of abuse and neglect in childhood may cause alterations in the functioning of the Hippocampal-Pituitary-Adrenal axis (Heim *et al*., 2001; De Bellis and A.B., 2014), which can then, in turn, increase reactivity to future, but still earlier, life stress (Heim *et al*., 2004). Therefore, it seems that childhood trauma experiences likely lead to maladaptive personality traits, impulsivity, and/or poor decision-making patterns (Allen *et al*., 2013; Braquehais *et al*., 2010; Huh *et al*., 2016) that are linked to early-onset suicidal behavior (Perry *et al*., 2021; Szücs *et al*., 2020). In contrast, these risk factors that are associated with earlier onset suicidal behavior may not have the same salience in predicting late-onset attempts. While previous research has found childhood abuse to be most strongly linked to early-onset depression, it also has implications for mid- and late-onset depression (Wielaard *et al*., 2018). Our findings that late-onset suicide attempters had similar levels of childhood trauma as other depressed groups suggest that childhood trauma does not play a prominent role in triggering late-onset suicidal crises, despite possibly contributing to depression in older populations.

As late-onset attempters are most similar to the population of older adults who may be more likely to die by suicide after only a single late-life attempt (Paraschakis *et al*., 2012; Perry *et al*., 2021), we still need much more research investigating how late-life suicidal behavior manifests and how it can be prevented. Our past research suggests that it is likely that cognitive deficits associated with aging may play a role (Gujral *et al*., 2020).

In addition to investigating the relationship between childhood trauma and onset of suicidal behavior, we also investigated how childhood trauma may relate to suicidal behavior. While we did not find early-onset attempters to have higher levels of impulsivity or maladaptive personality traits, they had a younger age of onset of depression and PTSD and scored higher on both lifetime and current (late-life) diagnosis of PTSD. This is consistent with other studies finding a relationship between childhood trauma and future PTSD, as early negative life experiences can be reactivated by stressful life events occurring years after the initial trauma (Maschi *et al*., 2013). In addition, as the early-onset attempters in our study still experienced suicidal ideation, and sometimes even suicidal behavior, into late life, while these experiences of childhood trauma may have early consequences, the experience of childhood trauma can also have negative emotional and behavioral ramifications all the way into late life. While our finding that early-onset attempters had similar levels of impulsivity and borderline traits as late-onset attempters was unexpected, previous research has shown that borderline personality disorder tends to “burn out” with age, and older individuals with this disorder exhibit less impulsivity than their younger counter-parts (Stevenson *et al*., 2003). Moreover, our earlier study (Buerke *et al*., 2021) found that while impulsivity and borderline traits follow this age-related decline, they are still higher in suicide attempters as compared to depressed non-attempters.

Furthermore, our finding that emotional abuse and neglect were highest in early-onset attempters is consistent with past findings that show emotional abuse as the childhood trauma subtype most consistently related to forms of internalizing and externalizing psychopathology and findings that emotional abuse and neglect commonly co-occur (Cecil *et al*., 2017). Our results that indicated physical neglect was higher in early-onset attempters than most other groups are particularly novel, as most studies have not found a significant relationship between physical neglect and suicide risk in any age group. We also found physical abuse to be higher in early-onset attempters than in all comparison groups, but not late-onset attempters. This is interesting because physical abuse has been consistently related to suicidal behavior (i.e., Behr Gomes Jardim *et al*., 2018). However, it is possible that the ramifications of experiencing such abuse may not be limited to a particular timepoint (i.e., suicidal behavior early or late in life), and this is why we did not observe differences in the timing of that suicidal behavior.

Lastly, while sexual abuse has been consistently related to suicidal behavior, especially in males (Duke *et al*., 2010; Martin *et al*., 2004), our study did not find any group differences in sexual abuse. This is consistent with prior research on low rates of endorsement of sexual childhood trauma in unipolar depression in adulthood (Zhang *et al*., 2020). In addition, our sample’s low endorsement rates of physical and sexual abuse across groups may have underpowered us to detect differences between groups.

## Strengths and limitations

### Strengths:

Our case-control design allowed us to compare the relationship between childhood abuse and age of onset of suicidal behavior, over and above its relationship with ideation and depression. In contrast to previous studies restricting age of onset to young adulthood, our late-life sample provided the opportunity to examine onset of suicidal behavior in those who are still suicidal well into late life. This study also improved upon the traditional median cutoff for identification of age of onset of suicide attempt (Gujral *et al*., 2020; Kenneally *et al*., 2019; Szücs *et al*., 2020; Perry *et al*., 2021) by applying GMM, a more data-driven approach that has been applied in one other study (Slama *et al*., 2009), to identify different types of attempters.

### Limitations:

As age of first attempt and childhood trauma experiences were assessed retrospectively, recall bias may have occurred. Additionally, as the CTQ-SF is a self-report, participants may have withheld answers they felt uncomfortable disclosing. This may be especially important for findings related to sexual abuse, as research suggests that older adults, particularly male, are less likely to disclose sexual trauma experiences (Bows, 2018; O’Leary and Barber, 2008; Stemple and Meyer, 2014). Furthermore, it is important to acknowledge that our study involved multiple comparisons (see Table 3), which increases the likelihood of Type I error. However, as we focused on examining the association between childhood trauma and suicide, our interest in potential pathways for this relationship was secondary, and we believe that these analyses contribute to the comprehensive understanding of the relationship between childhood trauma and late-life suicide.

Importantly, our results may be subject to survival bias, as we can only retrospectively study childhood trauma experiences in older adults who have survived their suicidal behaviors; therefore, those who experienced more severe childhood trauma and enacted lethal suicidal behavior would be absent from our study. This may also partially explain the smaller number of early-onset attempters in our study of late-life suicidal behavior.

## Conclusions and clinical implications

There is substantial heterogeneity among depressed older adults who attempt suicide and experience suicidal ideation in late life. Our findings suggest that childhood trauma contributes to suicide risk in a specific subset of suicidal elderly. Particularly we found that childhood trauma is significantly associated with early-onset suicidal behavior in late-life depression. Our finding emphasizes the importance of early intervention and prevention efforts to mitigate the impact of childhood adversity. Clinicians should be aware of differences in risk factors (e.g., childhood trauma, personality traits, familial risk, decision-making) and trajectories (e.g., a greater number of less lethal attempts) associated with early-onset suicidal behavior and intervene accordingly. Similarly, clinicians should be aware of the unique risk factors associated with late-onset suicide attempts, including cognitive impairment and late-onset depression. Overall, this study highlights the need for a personalized, multidimensional approach to suicide prevention in depressed older adults, taking into account individual risk factors and clinical characteristics.

## Figures and Tables

**Table 1.** Demographic characteristics by group (*N* = 224)

	early-onset attempters (eo)^1^ *N* = 28	late-onset attempters (lo)^1^ *N* = 56	ideators (ide) *N* = 44	depressed comparison group (DC) *N* = 58	healthy comparison group (HC) *N* = 38
**Age**	60.04 (5.67)	63.95 (7.87)	61.61 (6.69)	62.74 (7.46)	62.89 (8.16)
**Gender (female)**	22 (78.57%)	28 (50.00%)	23 (52.27%)	33 (56.90%)	22 (57.89%)
**Race**					
** Caucasian**	21 (75.00%)	47 (83.93%)	39 (88.64%)	46 (79.31%)	35 (92.11%)
** Black or African American**	7 (25.00%)	6 (10.71%)	3 (6.82%)	11 (18.97%)	3 (7.89%)
** Asian**	0 (0.00%)	1 (1.79%)	1 (2.27%)	1 (1.72%)	0 (0.00%)
** More than One Race**	0 (0.00%)	2 (3.57%)	1 (2.27%)	0 (0.00%)	0 (0.00%)
**Ethnicity**	2.00 (0.00)	1.96 (0.19)	2.00 (0.00)	2.00 (0.00)	2.00 (0.00)
**Education**	14.71 (2.73)	13.82 (2.46)	15.16 (2.25)	15.52 (2.55)	16.13 (2.60)
**Income (per capita)**	17,397.44 (11,406.27)	20,999.23 (12,306.81)	26,516.47 (19,047.84)	22,841.52 (17,659.08)	39,281.54 (21,221.52)

1 Attempters were separated into early and late-onset based on age of first attempt using a statistical algorithm that identified a cutoff age of 30 years old.

**Table 2.** Childhood trauma experience comparison by group (*N* = 224)

	early-onset attempters^1^ *N* = 28 M (SD) or n (%)	late-onset attempters^1^ *N* = 56 M (SD) or n (%)	ideators *N* = 44 M (SD) or n (%)	depressed comparison group *N* = 58 M (SD) or n (%)	healthy comparison group *N* = 38 M (SD) or n (%)	p-value	post-hoc comparison
**Emotional Abuse**	14.29 (5.26)	10.21 (5.66)	10.98 (5.84)	9.91 (4.41)	6.13 (2.20)	<0.001	EO > LO&IDE&DC > HC
**Physical Abuse**	9.75 (3.85)	8.27 (3.82)	6.98 (2.82)	7.16 (2.61)	5.71 (1.33)	<0. 001	EO > IDE&DC&HC; LO > HC
**Sexual Abuse**	8.82 (6.68)	8.27 (5.69)	7.39 (5.54)	6.28 (3.07)	5.58 (2.45)	0.016	No pairwise differences
** For Male**	6.83 (3.60)	6.39 (3.83)	6.67 (4.94)	5.68 (1.46)	5.00 (0.00)	0.517	
** For Female**	9.36 (7.27)	10.14 (6.64)	8.04 (6.07)	6.73 (3.83)	6.00 (3.18)	0.044	No pairwise differences
**Emotional Neglect**	15.79 (5.50)	11.62 (5.49)	11.48 (5.98)	11.26 (4.87)	7.79 (3.24)	<0.001	EO > LO&IDE&DC > HC
**Physical Neglect**	9.57 (3.55)	7.62 (3.09)	7.55 (3.09)	7.41 (2.69)	5.82 (1.86)	<0.001	EO > LO&IDE&DC&HC; LO > HC
**CTQ-SF Total** ^2^	49.68 (14.47)	38.02 (15.97)	37.14 (16.09)	35.91 (11.46)	25.50 (6.64)	<0.001	EO > LO&IDE&DC > HC
**At Least One form of Trauma**	24 (85.71%)	31 (55.36%)	18 (40.91%)	32 (55.17%)	8 (21.05%)	<0.001	EO > LO&IDE&DC&HC; LO&DC > HC
**Multiple forms of Trauma**	19 (67.86%)	21 (37.50%)	13 (29.55%)	18 (31.03%)	1 (2.63%)	<0.001	EO > LO&IDE&DC > HC

1 Attempters were separated into early and late-onset based on age of first attempt using a statistical algorithm that identified a cutoff age of 30 years old.

2 Childhood Trauma Questionnaire – Short Form (CTQ-SF).

**Table 3.** Clinical characteristics by group (*N* = 224)

	early-onset attempters (eo)^1^ *N* = 28	late-onset attempters (lo)^1^ *N* = 56	ideators (ide) *N* = 44	depressed comparison group (DC) *N* = 58	healthy comparison group (HC) *N* = 38	p-value	post-hoc comparison
**Borderline Traits** ^2^	32.81 (11.07)	30.89 (15.90)	32.16 (11.81)	25.58 (9.59)	8.86 (5.29)	<0. 001	EO&LO&IDE&DC > HC; IDE > DC
**Impulsivity** ^3^	69.96 (10.10)	69.61 (12.87)	67.77 (10.27)	65.31 (10.51)	53.62 (8.23)	<0001	EO&LO&IDE&DC > HC
**Dementia Rating Scale Total Score** ^4^	135.27 (4.63)	134.72 (4.96)	136.69 (4.45)	136.33 (4.56)	138.76 (2.85)	0.002	EO&LO < HC
**Executive Function** ^5^	6.74 (3.23)	7.63 (3.19)	6.24 (3.18)	6.02 (3.59)	5 .13 (2. 68)	0.016	LO > HC
**Mental Status Exam** ^6^	28.25 (1.33)	28.18 (1.80)	28.88 (1.30)	28.96 (0.93)	29.30 (0.88)	<0.001	EO&LO < HC; LO < DC
**Lifetime substance abuse**	17 (62.96%)	31 (59.62%)	23 (52.27%)	25 (43.86%)	–	0.272	
**Current substance abuse**	4 (14.81%)	8 (15.38%)	1 (2.27%)	7 (12.28%)	–	0.120	
**Lifetime anxiety**	24 (88.89%)	39 (75.00%)	35 (79.55%)	32 (56.14%)	–	0.006	EO > DC
**Current anxiety**	20 (74.07%)	34 (65.38%)	29 (65.91%)	27 (47.37%)	–	0.064	
**Lifetime PTSD**	16 (61.54%)	11 (21.57%)	10 (22.73%)	5 (8.77%)	–	<0.001	EO > LO&IDE&DC
**Current PTSD**	10 (38.46%)	5 (9.80%)	8 (18.18%)	4 (7.02%)	–	0.003	EO > LO&DC
**Depression Severity (no suicide item)** ^7^	21.15 (4.87)	19.82 (6.18)	19.26 (5.42)	17.02 (4.07)	–	0.003	EO&LO > DC
**Age of Depression Onset**	16.64 (7.99)	42.61 (18.44)	37.64 (17.45)	42.08 (17.08)	–	<0.001	EO < LO&IDE &DC
**Current Suicide Ideation** ^8^	16.48 (8.40)	23.02 (7.82)	16.20 (5.71)	–	–	<0.001	LO > EO&IDE
**Worst Lifetime Suicide Ideation** ^8^	24.70 (6.43)	26.31 (5.24)	20.23 (6.99)	–	–	<0.001	EO&LO > IDE
**Age of First Suicide Attempt**	17.50 (6.35)	57.16 (12.39)	–	–	–	<0.001	EO < LO
**Highest lethality suicide attempt**	3.11 (2.34)	3.52 (1.83)	–	–	–	0.390	
**Lifetime number of suicide attempts**	2.71 (1.74)	1.61 (0.95)	–	–	–	<0.001	EO > LO
**Worst lifetime intent - planning subscore** ^9^	7.50 (2.00)	7.93 (3.20)	–	–	–	0.546	
**Worst lifetime intent - total score** ^9^	17.43 (4.35)	18.68 (5.13)	–	–	–	0.311	

1 Attempters were separated into early and late-onset based on age of first attempt using a statistical algorithm that identified a cutoff age of 30 years old.

2 Personality Assessment Inventory – Borderline subscale (PAI-BOR).

3 Barratt Impulsiveness Scale (BIS-11).

4 Dementia Rating Scale (DRS).

5 Executive Interview (EXIT).

6 Mini-Mental State Examination (MMSE).

7 Hamilton Rating Scale for Depression – 17 Item (HRSD).

8 Scale of Suicidal Ideation (SSI).

9 Scale of Suicidal Intent (SIS).
